# Nanomechanical properties of enucleated cells: contribution of the nucleus to the passive cell mechanics

**DOI:** 10.1186/s12951-020-00696-1

**Published:** 2020-09-17

**Authors:** Yuri M. Efremov, Svetlana L. Kotova, Anastasia A. Akovantseva, Peter S. Timashev

**Affiliations:** 1 Institute for Regenerative Medicine, Sechenov University, 8 Trubetskaya St., Moscow, 119991 Russia; 2 N.N. Semenov Institute of Chemical Physics, 4 Kosygin St., Moscow, 119991 Russia; 3 Institute of Photon Technologies of Federal Scientific Research Centre “Crystallography and Photonics” of Russian Academy of Sciences, Pionerskaya 2, Troitsk, Moscow, 108840 Russia; 4 Chemistry Department, Lomonosov Moscow State University, Leninskiye Gory 1-3, Moscow, 119991 Russia

**Keywords:** Nucleus, Enucleated cells, Cytoplasts, AFM, Cell mechanical properties, Viscoelasticity

## Abstract

**Background:**

The nucleus, besides its functions in the gene maintenance and regulation, plays a significant role in the cell mechanosensitivity and mechanotransduction. It is the largest cellular organelle that is often considered as the stiffest cell part as well. Interestingly, the previous studies have revealed that the nucleus might be dispensable for some of the cell properties, like polarization and 1D and 2D migration. Here, we studied how the nanomechanical properties of cells, as measured using nanomechanical mapping by atomic force microscopy (AFM), were affected by the removal of the nucleus.

**Methods:**

The mass enucleation procedure was employed to obtain cytoplasts (enucleated cells) and nucleoplasts (nuclei surrounded by plasma membrane) of two cell lines, REF52 fibroblasts and HT1080 fibrosarcoma cells. High-resolution viscoelastic mapping by AFM was performed to compare the mechanical properties of normal cells, cytoplasts, and nucleoplast. The absence or presence of the nucleus was confirmed with fluorescence microscopy, and the actin cytoskeleton structure was assessed with confocal microscopy.

**Results:**

Surprisingly, we did not find the softening of cytoplasts relative to normal cells, and even some degree of stiffening was discovered. Nucleoplasts, as well as the nuclei isolated from cells using a detergent, were substantially softer than both the cytoplasts and normal cells.

**Conclusions:**

The cell can maintain its mechanical properties without the nucleus. Together, the obtained data indicate the dominating role of the actomyosin cytoskeleton over the nucleus in the cell mechanics at small deformations inflicted by AFM. 
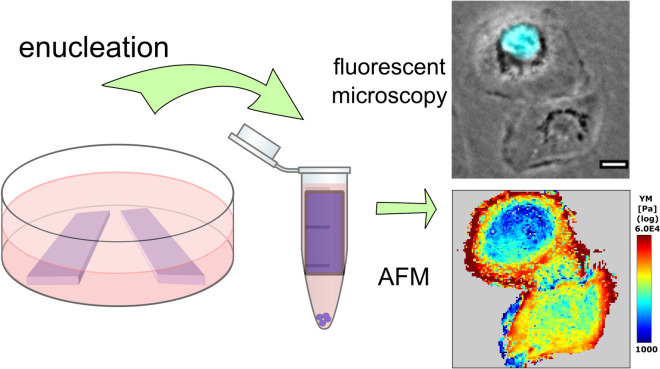

## Introduction

The nucleus is the largest cellular organelle whose main well-known functions include DNA replication and gene regulation. At the same time, the nucleus continually resists and responds to external and intercellular mechanical forces and plays a significant role in mechanosensitivity and mechanotransduction [1–4]. A proper genome organization and expression are dependent on the nuclear mechanics, and its alterations are associated with many human diseases, including progeria, muscular dystrophies, and cancer [5–8].

The nanomechanical properties of the nucleus were analyzed in a number of previous studies. While quite often the nucleus is considered to be the stiffest organelle in the cell [1, 9–12], other studies have shown that the nucleus is relatively soft, at least softer than the cytoskeletal structures [6, 13–15]. The recent studies have established that the mechanical behavior of the nucleus is quite complex, and different properties are expected in different deformation ranges and modes [3, 16, 17]. The mechanical properties of the nucleus were shown to be determined by chromatin at low deformations, and by lamina (a meshwork of intermediate filaments under the nuclear envelope) at high deformations [16, 17]. Furthermore, inside the cell, its mechanical properties are modulated by interactions with the cytoskeleton [13, 18, 19] and the active transport imbalance across the nuclear envelope [20]. The nuclear mechanics is cell-type-specific [21, 22] and significantly evolves during many normal processes such as differentiation and aging, and under pathological conditions [6, 12].

To better understand the role of the nucleus in different processes, a cellular enucleation approach has been used to explore different cellular properties in the absence of the nucleus [23–25]. Surprisingly, the studies have discovered that the nucleus might be dispensable for many cellular activities, like polarization and migration in 1D and 2D [26, 27]. However, the nucleus was required for the proper cell mechanical responses and 3D migration. How the mechanical properties of the cell itself are affected by the absence of the nucleus has not been investigated yet. Here, we utilized the enucleation approach to assess the mechanical properties of enucleated cells (cytoplasts), and also to compare them with the properties of isolated nuclei (nucleoplasts) obtained by the same and other techniques. Two cell lines, REF52 rat fibroblasts and HT1080 human fibrosarcoma cells, were used for such a comparison.

## Material and methods

### Cell cultures and enucleation

Two cell lines were used in the study, REF52 rat fibroblasts and HT1080 human fibrosarcoma cells. The cells were cultured in the DMEM medium supplemented with 1 × GlutaMax, 15 mM HEPES, 10% FBS and 1% antibiotic/antimycotic solution (all by Thermo Fisher, USA) in a humidified 5% CO_2_ atmosphere at 37 °C.

**Fig. 1:**
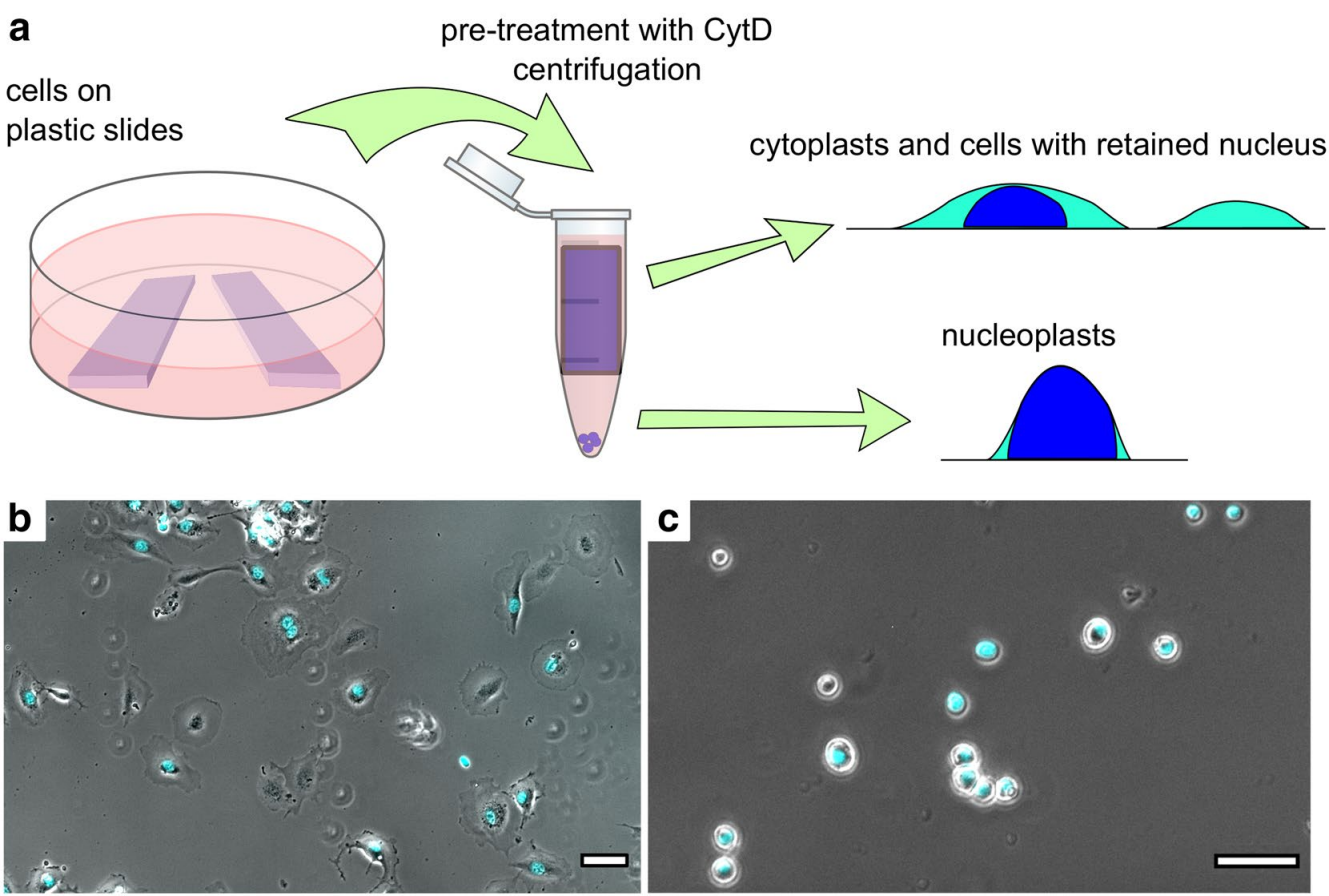
The scheme of the enucleation protocol. **a** Cells growing on plastic slides were inserted in 1.5 mL Eppendorf tubes filled with the cell medium and pre-treated with CytD for 15 min. After the centrifugation, the remaining cells and cytoplasts were reseeded from the plastic slides to fibronectin-coated Petri dishes, and AFM experiments were performed 3 to 5 h after. The pellet containing nucleoplasts was resuspended in HBSS and placed on poly-L-lysine-coated dishes for further experiments. **b** A sample of REF52 enucleated cells contained both cells without the nucleus (enucleated cells, cytoplasts) and cells with the retained nucleus. The cell-permeable DNA dye (cyan) was used to distinguish cytoplasts from normal cells. **c** Nucleoplasts. DNA staining was used to distinguish nucleoplasts from the cell debris. Scale bars are 50 µm

Enucleation was performed by the previously described approach [23–25] with some modifications (Fig. 1). Small pieces of culture plastic (plastic slides) with an approximate size of 8 × 20 mm were cut from standard cell culture Petri dishes (Corning, USA). The plastic slides were sterilized by incubation in 95% ethanol and exposed to a germicidal UV lamp (Microcide, Electronic Medicine, Russia) for 10 min, then coated with a 10 µg/mL fibronectin (Sigma, USA) solution for 20 min. Cells growing as monolayers on plastic slides were inserted in 1.5 mL Eppendorf tubes filled with 2 µM of Cytochalasin D (CytD) (Sigma, USA) in the cell medium. After 15 min of pre-incubation at 37 °C, the cells were centrifuged at 13,400 rpm (12,000 g) in a MiniSpin microcentrifuge (Eppendorf, Germany) for 9 to 25 min (longer duration needed for REF52 cells). After centrifugation, the cells on the plastic slides were washed with PBS, trypsinized, and reseeded on fibronectin-coated glass-bottom cell culture dishes (WillCo Wells B.V., Amsterdam, Netherlands). After 3 to 5 h, the cells (including cytoplasts) were fully spread and recovered from the effects of CytD. The medium was exchanged for the fresh one before the AFM experiment. The pellet containing nucleoplasts was resuspended in HBSS (Hanks balanced salt solution, Thermo Fischer, USA) and placed on poly-L-lysine-coated glass-bottom dishes. After 15 min of incubation, unattached nucleoplasts were removed by washing with HBSS.

The nuclei were also isolated using a detergent-based approach. Briefly, the cells were transferred to suspension by trypsinization, centrifuged and resuspended in a 0.1% Triton X-100 detergent (Thermo Fischer, USA) solution in HBSS, mildly pipetted and centrifuged for 10 s at 12,000 *g*. The detergent treatment was performed twice, on ice with ice-cold solutions to minimize the nuclei’s damage and degradation due to cellular proteases. Then the pellet was resuspended in HBSS and a small amount of the solution was transferred to poly-L-lysine-coated glass-bottom dishes for AFM experiments. After 15 min of incubation, the unattached nuclei were removed by washing with HBSS.

### Atomic force microscopy

All AFM measurements were performed using a Bioscope Resolve AFM (Bruker, Santa Barbara, USA) mounted on an Axio Observer inverted fluorescent microscope (Carl Zeiss, Germany). The microscope was equipped with a heated stage, and the sample temperature was kept constant at 37 °C during the experiments with cells. PeakForce QNM-Live Cell probes (PFQNM-LC-A-CAL, Bruker AFM Probes, Camarillo, CA, USA), short paddle-shaped cantilevers with a pre-calibrated spring constant (values were in the range of 0.06–0.08 N/m) were used. The cantilever deflection sensitivity (nm/V) was calibrated from the thermal spectrum directly in the dish with a sample using the pre-calibrated value of the spring constant [28]. The nanomechanical and topography maps were acquired in the fast force volume (FFV) mode with a map size from 40 × 40 to 100 × 100 µm and from 32 × 32 to 128 × 128 point-measurements. The force curves (*F-Z* curves) had a vertical ramp distance of 3 μm, a vertical piezo speed of 183 μm/s, and the trigger force of 0.5–1 nN. The topography and local height were calculated from the force curves by the contact point position versus contact position over the substrate, the global tilt correction was performed if needed.

The numerical processing of the *F-Z* curves was done using MATLAB scripts (The MathWorks, Natick, MA) developed in the previous works [29, 30] with the utilization of the Ting’s model [31]:

1 $$F\left( {t,\delta \left( t \right)} \right) = \frac{4\sqrt R }{{3\left( {1 - \nu^{2} } \right)}}\mathop \smallint \limits_{0}^{{t_{1} \left( t \right)}} f_{BEC} (\delta )E\left( {t - \xi } \right)\frac{{\partial \delta^{\frac{3}{2}} }}{\partial \xi }d\xi ;$$

2 $$t_{1} \left( t \right) = \left\{ {\begin{array}{*{20}c} {t_{1} \left( t \right) = t, 0 \le t \le t_{m} } \\ {\mathop \smallint \limits_{{t_{1} \left( t \right)}}^{t} E\left( {t - \xi } \right)\frac{\partial \delta }{{\partial \xi }}d\xi = 0, t > t_{m} } \\ \end{array} } \right.;$$

where *F* is the force acting on the cantilever tip; $\delta$ is the indentation depth; $t$ is the time initiated at the contact; $t_{m}$ is the duration of the approach phase; $t_{1}$ is the auxiliary function determined by Eq. 2; $\xi$ is the dummy time variable required for the integration; $\nu$ is the Poisson’s ratio of the sample (assumed to be time-independent and equal to 0.5); $R$ is the radius of the indenter; $f_{BEC} (\delta )$ is the bottom-effect correction factor [32]; and $E(t)$ is the Young’s relaxation modulus for the selected rheology model. Here we used the power-law rheology (PLR) model (also known as a springpot in parallel with a dashpot) [33, 34]:

3 $$E\left( t \right) = E_{1} t^{ - \alpha } + \eta \delta_{D} (t),$$

where $E_{1}$ is the relaxation modulus at *t* = 1 s (scale factor of the relaxation modulus); $\alpha$ is the power-law exponent; $\eta$ is the Newtonian viscous term (with Pa*s units); and $\delta_{D} (t)$ is the Dirac delta function. A larger $\alpha$ value means a larger amount of relaxation; materials exhibit a solid-like behavior at $\alpha = 0$, and a fluid-like behavior at $\alpha = 1$. The PLR model described by Eq. [3] was successfully used for the description of cell mechanics in several previous studies [33, 35–37]. The Young’s modulus with the assumptions of the Hertz’s theory, YM (“apparent” elastic modulus), was also calculated from the approach part of the force curves [38].

We used the top 50% of each cell data set over a cell to define the nuclear part, and the lower areas were discarded in the analysis, since their local properties were highly affected by the high F-actin concentration at the periphery. The same part of the dataset was used for the nucleoplasts and isolated nuclei as well, to exclude the peripheral regions where the data can potentially be affected by the local tilt of the hemispherical sample. From the datasets, we used the mean geometric values of YM and $E_{1}$, and mean arithmetic values of $\alpha$ and $\eta$ for the further comparison between the samples [36]. In the text, the data are presented as mean ± SD. All the statistical analyses were performed using the MATLAB software (MathWorks, USA). A non-parametric Mann–Whitney U test was used to determine the statistically significant differences between the groups. The change in the parameters was calculated as that relative to the median value. The percentiles in the box-and-whisker plots are 10%, 25%, 50%, 75%, and 90%, the dots correspond to each value of the set.

### Fluorescent and confocal microscopy

The nuclei in the prepared AFM samples were stained with Hoechst 33342 dye (2 µg/mL) to detect enucleated cells or to distinguish the nucleoplasts and isolated nuclei from the cell debris. Phase-contrast and fluorescent images were recorded with 10x/0.3 or 20x/0.4 objectives using the ZEN software (Carl Zeiss, Germany) of the Axio Observer inverted fluorescent microscope.

For the confocal microscopy studies, the samples were fixed in a 4% formaldehyde solution in PBS for 10 min, permeabilized with 0.1% Triton X-100 for 10 min, blocked with 1% bovine serum albumin for 10 min and stained with Alexa Fluor 488 phalloidin (Life Technologies, USA). The samples were washed with PBS and mounted with the ProLong Gold antifade reagent (Invitrogen, USA). Fluorescent images (Z-stacks) were acquired using an LSM 880 confocal laser scanning microscope equipped with an AiryScan module and a GaAsP detector (Carl Zeiss, Germany) with a Plan-Apochromat 63x/1.4 N.A. oil immersion objective.

## Results

**Fig. 2:**
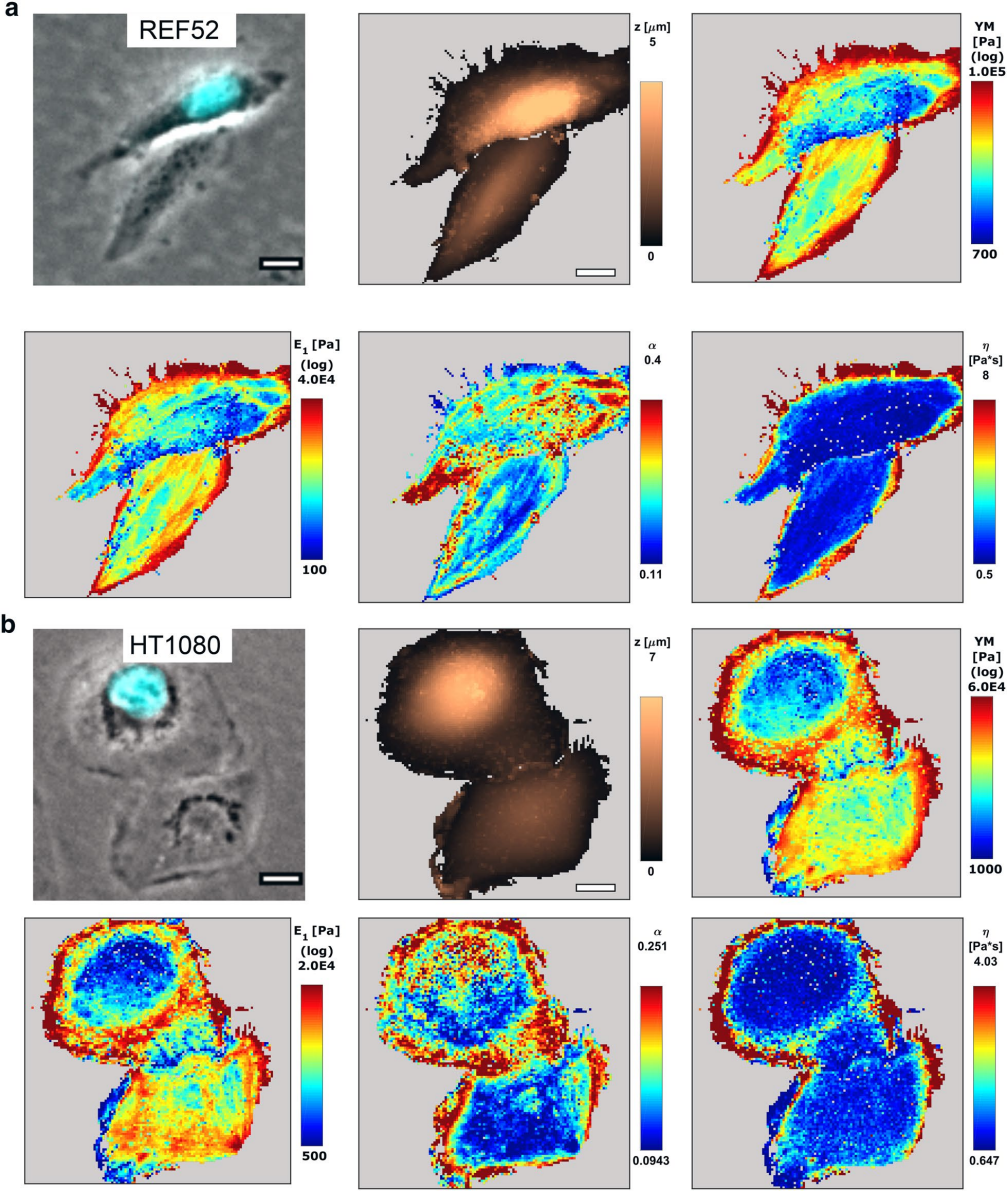
Examples of nanomechanical maps acquired over REF52 (**a**) and HT1080 (**b**) cells after the enucleation: the topography (z), apparent YM, and viscoelastic parameters ($E_{1}$, $\alpha$, $\eta$). A cytoplast and a cell with the retained nucleus are presented on the same map. The phase-contrast image is aligned with the fluorescence of the DNA stain (cyan). Cytoplasts have a lower height as seen on the topography (Z) and a higher stiffness (YM and $E_{1}$), while other viscoelastic parameters ($\alpha$, $\eta$) differ less. Scale bars are 20 µm on the optical images and 10 µm on the AFM maps

**Fig. 3:**
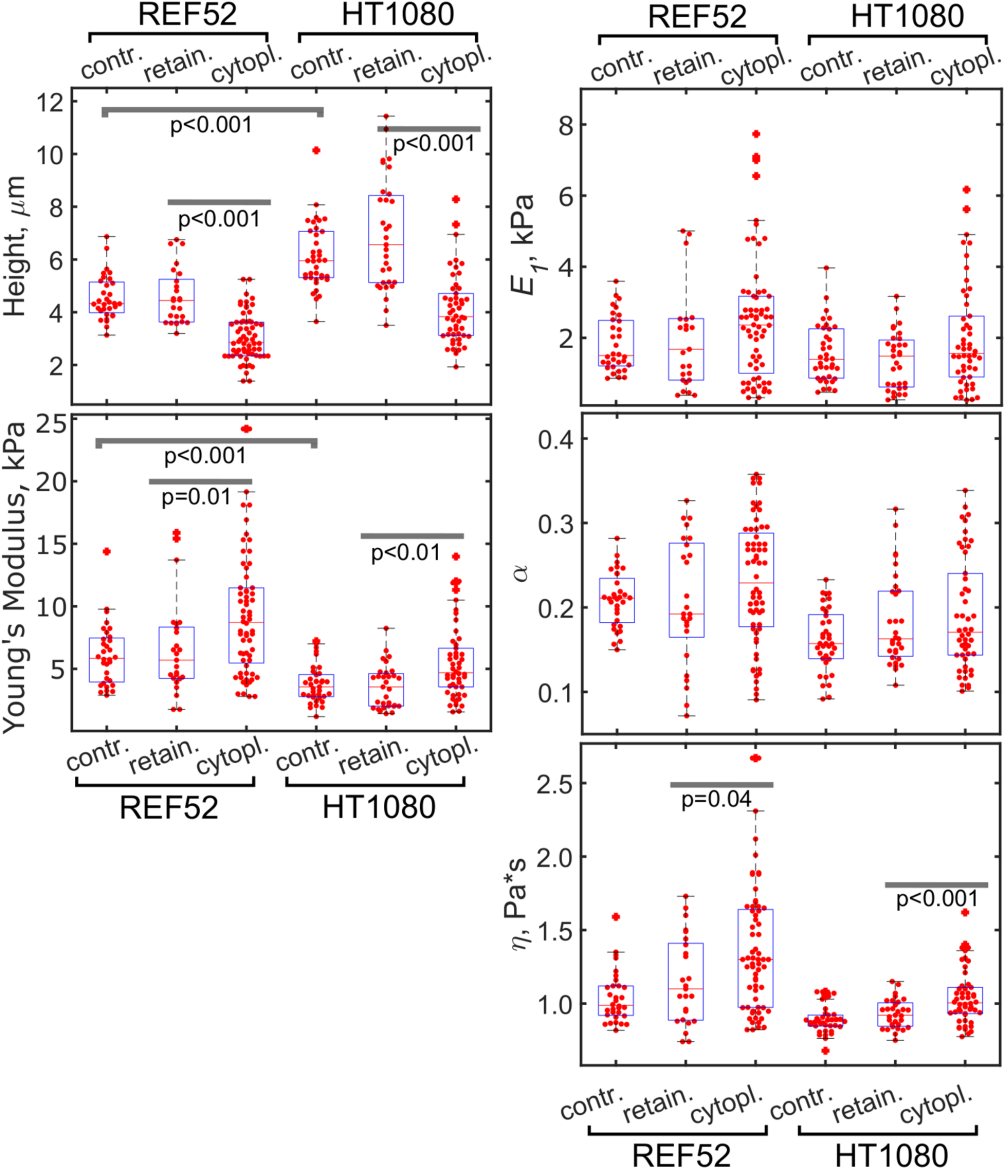
The height, apparent YM, and viscoelastic parameters ($E_{1}$, $\alpha$, $\eta$) of REF52 and HT1080 control cells, cells with the retained nucleus, and cytoplasts. Cytoplasts had a lower height, a higher stiffness (YM and $E_{1}$), and a higher apparent viscosity ($\eta$), while the control cells and cells with the retained nucleus had similar properties

To obtain cytoplasts and nucleoplasts, the enucleation protocol was adapted from the earlier studies [23–25]. The nuclei were removed from the cells with disrupted actin cytoskeleton by centrifugal forces. The protocol parameters (centrifugation time) were adjusted to achieve the enucleation efficiency around 50%. Notably, HT1080 cells with the less-developed actin cytoskeleton and lower stiffness, which agrees with their tumor origin [39], required milder conditions to achieve the same enucleation efficiency as that for REF52 fibroblasts. In this way, the resulted sample had both cytoplasts and cells with the retained nucleus, which served as an internal control (Figs. 1, 2). The presence or absence of the nucleus was confirmed both morphologically and by staining DNA with the Hoechst 33342 cell-permeant dye. The cells with the retained nucleus had similar morphology and mechanical properties (Fig. 3) as those for normal cells, proving that the enucleation procedure itself did not inflict a substantial cell damage, and that a complete cytoskeleton recovery also occurred after the procedure. The enucleated cells, as previously shown [26], were smaller and showed a substantial (by ~ 50%) height decrease (REF52: 3 ± 1 vs 4.5 ± 1 µm; HT1080: 4 ± 1 vs 7 ± 2 µm) as measured from the AFM force maps (Figs. 2, 3).

**Fig. 4:**
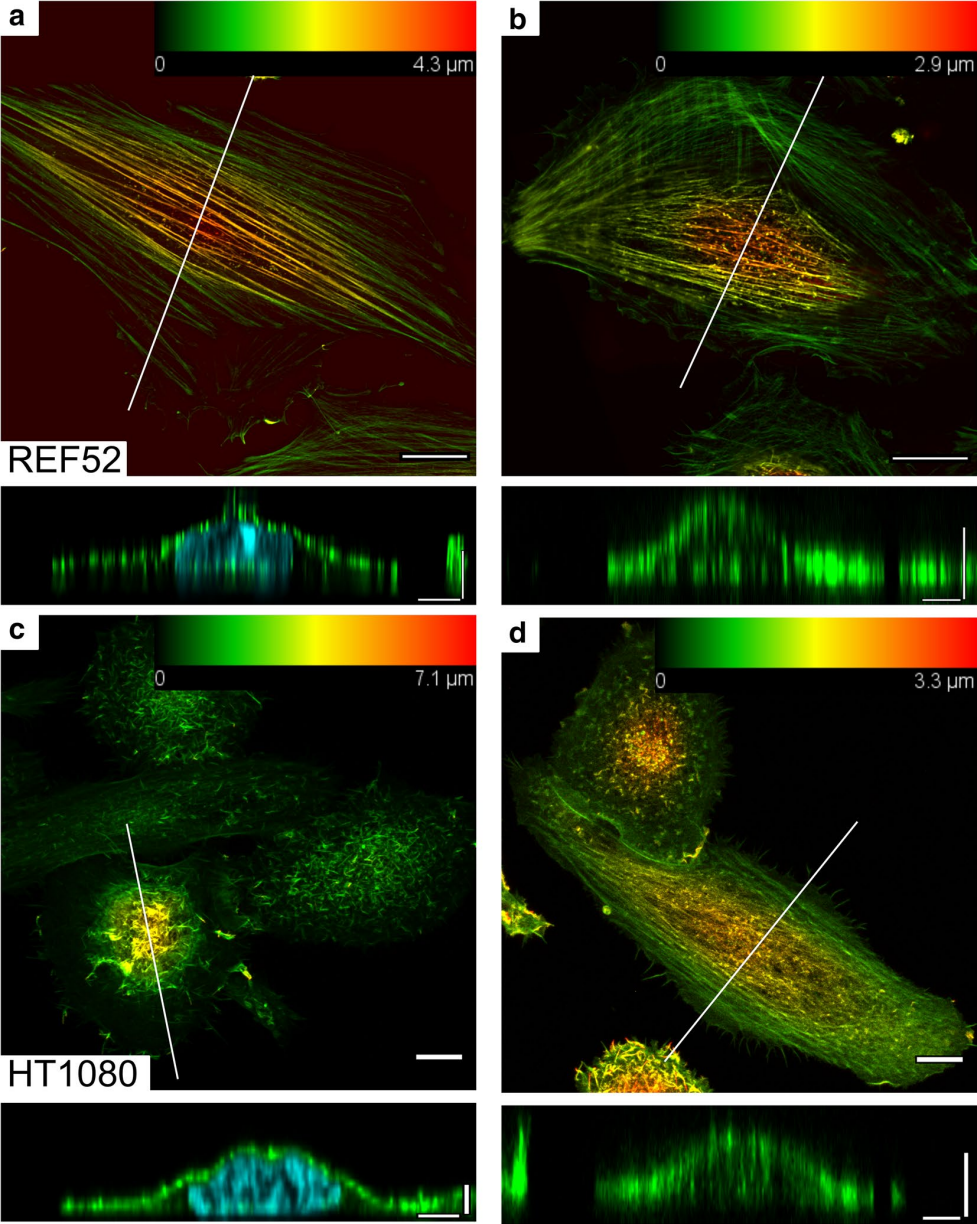
The actin cytoskeleton of the cells with the retained nucleus (**a**, **c**) and cytoplasts (**b, d**) of REF52 (**a, b**) and HT1080 (**c, d**) cells after the enucleation. Color-coded z-projections of the F-actin staining and reconstructed vertical cross-sections along the marked lines over cells and cytoplasts (green for F-actin, cyan for nucleus staining) are shown. Apical stress fibers that are going over the nucleus (over the nucleus-free cytoplasm in cytoplasts) could be identified in REF52 fibroblasts (**b**). Scale bars are 10 μm for the z-projections; 5 μm in the horizontal direction and 2 μm in the vertical direction for the cross-sections

Enucleation did not lead to a cell’s softening, as would be expected from the removal of the large stiff compartment (Fig. 3). Instead, a significant increase in the apparent YM was observed (REF52: 9 ± 5 vs 6 ± 4 kPa; HT1080: 5 ± 3 vs 3.6 ± 1.7 kPa). This increase, apparently, was not associated with the decrease in the cell height, since the bottom-effect correction was applied in the applied mechanical model [30]. Cytoplasts had a higher variability in the measured modulus, probably due to incomplete actin cytoskeleton recovery in some of them. The scale factor of the power-law rheology model $E_{1}$ followed the same trend as the YM did (REF52: 2.5 ± 1.8 vs 1.8 ± 1.4 kPa; HT1080: 2.0 ± 1.5 vs 1.4 ± 0.8 kPa), while the power-law exponent $\alpha$ did not change significantly (REF52: 0.23 ± 0.1 vs 0.21 ± 0.1; HT1080: 0.19 ± 0.1 vs 0.18 ± 0.1), and the apparent viscosity $\eta$ demonstrated an increase (REF52: 1.4 ± 0.4 vs 1.1 ± 0.3 Pa*s; HT1080: 1.1 ± 0.2 vs 0.9 ± 0.1 Pa*s) in enucleated cells. The last parameter indicates a stronger dissipative behavior in the absence of the nucleus. The actin cytoskeleton structure was checked with confocal microscopy on fixed samples and did not reveal substantial differences between cytoplasts and cells with the retained nucleus (Fig. 4). Surprisingly, at least in some of enucleated REF52 fibroblasts, the apical stress fibers similar to the perinuclear actin cap were found (Fig. 4b).

**Fig. 5:**
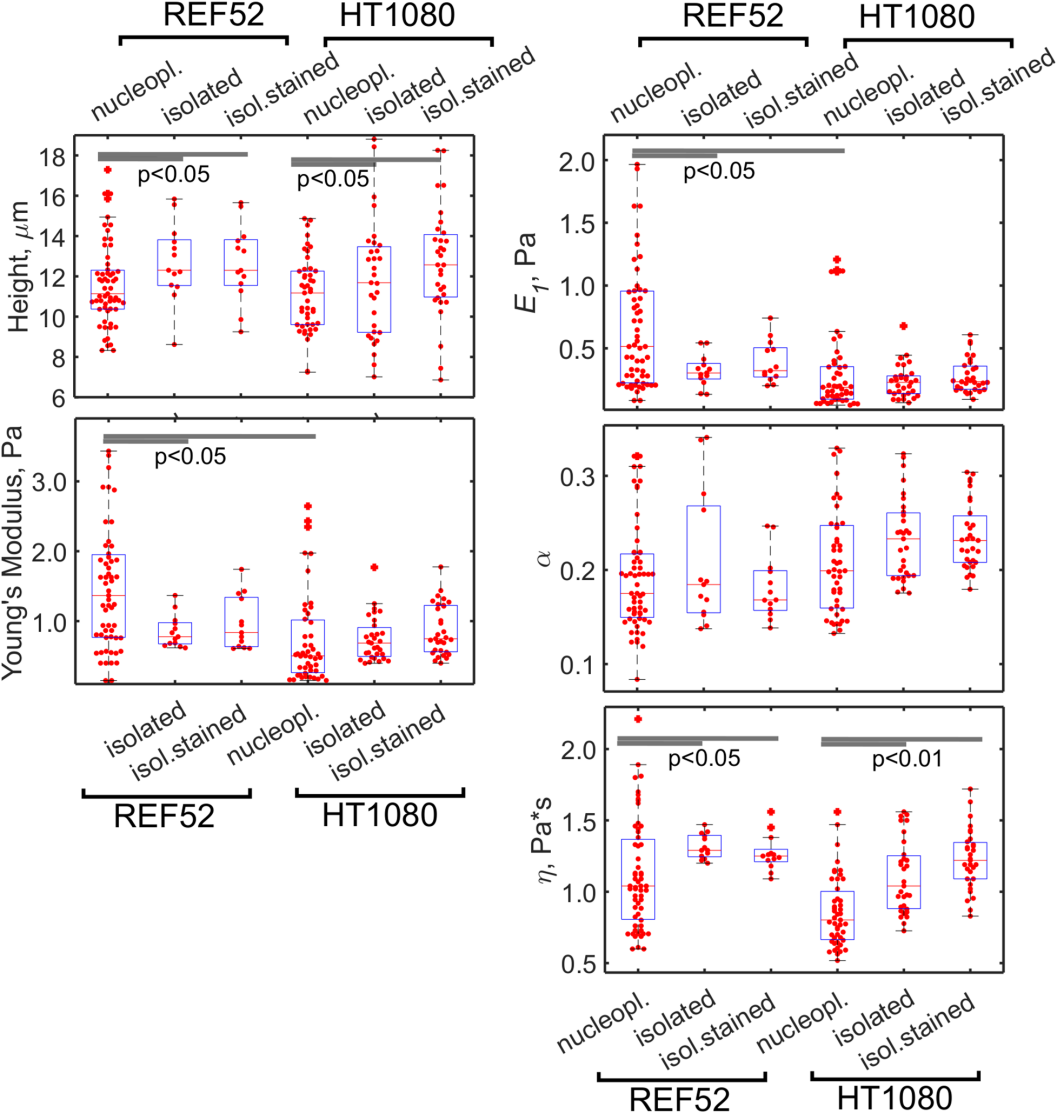
The height, apparent YM, and viscoelastic parameters ($E_{1}$, $\alpha$, $\eta$) of REF52 and HT1080 nucleoplasts and nuclei isolated with a detergent

**Fig. 6:**
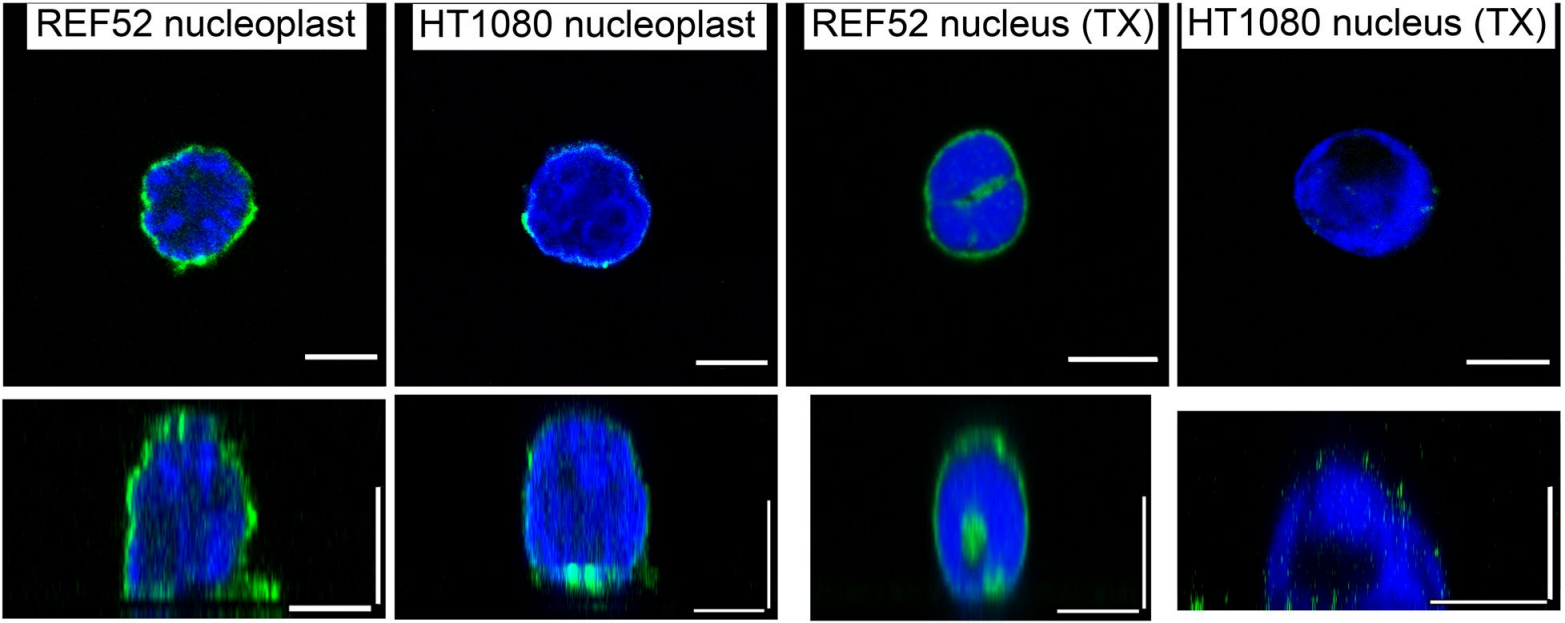
REF52 and HT1080 nucleoplasts and nuclei isolated with a detergent (TX). Z-sections over the middle plane and reconstructed vertical cross-sections along the nucleus center are shown, F-actin (green) and DNA staining (blue). Scale bars are 5 μm for the z-sections; 5 μm in the horizontal direction and 5 μm in the vertical direction for the cross-sections

The nucleoplast fraction that remained in the pellet after the enucleation procedure presents the nuclei surrounded by a small amount of the associated cytoplasm and covered by the plasma membrane. The Hoechst staining was used to distinguish nucleoplasts from the cellular debris. Nucleoplasts were much higher than the corresponding cells (REF52: 12 ± 2 vs 4.5 ± 1 µm; HT1080: 11 ± 2 vs 7 ± 2 µm) and were substantially softer than cells or cytoplasts for both cell types (the YM of REF52: 1.6 ± 1.1 vs 6 ± 4 kPa; the YM of HT1080: 0.7 ± 0.6 vs 3.6 ± 1.7 kPa), although the differences in the viscoelastic parameters did not show clear trends (Fig. 5). To check if the nucleus isolation protocol affects the measured properties, the nuclei were also isolated from cells using a detergent and mild pipetting at low temperatures. For HT1080 cells, the nuclei obtained by both isolation protocols had similar mechanical properties. But for REF52 fibroblasts, nucleoplasts were substantially stiffer than the nuclei obtained by the detergent method. Such a finding could be associated with some stiff residual F-actin in nucleoplasts, although some F-actin was found in the detergent-isolated nuclei as well (Fig. 6). A chromatin damage in the isolated nuclei cannot be completely excluded as well. To check if the chromatin plays a role in the measured mechanical properties, we compared the detergent-isolated nuclei with and without Hoechst staining. In the latter case, the measured YM was indeed slightly higher, although not significantly. The stiffening effect of the Hoechst staining was also observed in a previous study [17]. High-resolution force mapping revealed the presence of some stiffer structures inside the nuclei/nucleoplasts which could be associated with the areas of condensed chromatin or with the nucleoli [40] (Additional file 1. Fig. S1).

## Discussion

Although the nucleus is often considered as the stiffest cellular organelle, it might not be true for every situation. As was shown in the recent studies, the nuclear mechanics depends on the level of involved deformations [3, 16, 17]. At small deformations, the chromatin structure dominates the mechanical behavior. At large deformations, the lamina, located beneath the nuclear envelope, starts to play a more significant role. That is consistent with the observations of the current study, in which AFM inflicts only small deformations on the nucleus. Apparently, chromatin cannot provide the same high level of stiffness as the cortical actomyosin cytoskeleton is providing. However, the euchromatin/heterochromatin level was shown to modulate the nuclear stiffness, with increased heterochromatin leading to stiffening [15, 41]; and mutations in the lamina did not affect the global cell mechanical properties when measured by AFM [6].

The nanomechanical mapping performed here and by other groups [6, 41–43] has shown that the nuclear region is not stiffer than the other cell regions (Fig. 2). Moreover, the isolated nuclei appear softer than cells or cytoplasts when measured by routine AFM indentations in the low force/deformation mode. The lower-than-cytoskeleton nuclear stiffness was shown previously using a special needle-like AFM probe that penetrated the cell membrane and then deformed the nucleus directly [13]. However, a high stiffness might be observed at large deformations due to the lamina properties. Large deformations might be especially important for specific tissue types (e.g. muscular) and cells during 3D migration through narrow pores [44].

In a living cell, the nuclear shape and properties are also greatly modulated by the surrounding cytoskeleton [4, 45–47], and the reciprocal modulation might be also expected. The nucleus is compressed by the actin cytoskeleton, as might be seen for the low cell height in comparison with the isolated nucleus (Figs. 3 and 5). One of the theories explaining the origin of the cellular stiffness, the tensegrity model, describes the whole cellular structure as the pre-stressed scaffold, where the tensional forces originate from the actomyosin cytoskeleton and the compressional forces are exerted on the nucleus, microtubules, and other intracellular elements [19, 48]. It is hard to predict how the removal of such a large element as the nucleus will affect the balance of forces. The experiments conducted here, however, demonstrate that the cytoplast stiffness is actually increased in comparison with the normal cell, meaning that the cytoskeleton tension is redistributed on the remaining cellular structures (Fig. 3).

The actin cytoskeleton structure itself might be dependent on the direct linkage with the nucleus, such as that through the linker of nucleoskeleton-to-cytoskeleton (LINC) complexes [45, 49]. Mutations in the named complex often cause manifestation in the cytoskeleton appearance, and the presence of the special type of actin stress fibers, perinuclear actin cap, was shown to depend on the functional LINC complexes [47]. A disruption of such complexes had led to the disappearance of the actin cap, which was shown to be associated with the high cellular stiffness [50, 51]. Interestingly, here we discovered the arc-shaped stress fibers located in the same apical region where the perinuclear actin cap fibers might be located. The exact nature of these stress fibers was not established in the current study. Nevertheless, a significant disruption in the actin cytoskeleton would cause a decrease in the stiffness, while the opposite trend was observed here, indicating that the overall actin cytoskeleton structure was not adversely affected by the absence of the nucleus.

To conclude, we have shown that the cell can maintain its mechanical properties without the nucleus, at least at small deformations. Thus, the maintenance of the mechanical properties may be added to the list of the other functions weakly affected by the absence of the nucleus, like 1D and 2D migration. Nucleoplasts and the isolated nuclei were shown to be relatively soft in the same small deformation mode.

## Supplementary information

**Additional file 1:** **Figure S1.** Examples of nanomechanical maps acquired over REF52 (**a**) and HT1080 (**b**) nucleoplasts: the topography (z), apparent YM, and viscoelastic parameters ($E_{1}$, $\alpha$, $\eta$).

## Data Availability

The datasets used and/or analyzed during the current study are available from the corresponding author on reasonable request.
