# Supplementary material for: Nanomechanical properties of enucleated cells: contribution of the nucleus to the passive cell mechanics
Source: J Nanobiotechnology. 2020 Sep 17;18:134. doi: 10.1186/s12951-020-00696-1 (PMC7500557; doi:10.1186/s12951-020-00696-1)
Supplement: Supplementary file 1 — Additional file 1: Figure S1. Examples of nanomechanical maps acquired over REF52 (a) and HT1080 (b) nucleoplasts: the topography (z), apparent YM, and viscoelastic parameters ($E_{1}$E1, $\alpha$α, $\eta$η). [file 12951_2020_696_MOESM1_ESM.pdf]

Supplementary data

Nanomechanical properties of enucleated cells: contribution of the nucleus to the passive cell mechanics

Yu.M. Efremov, S.L. Kotova, A.A. Akovantseva, P.S. Timashev

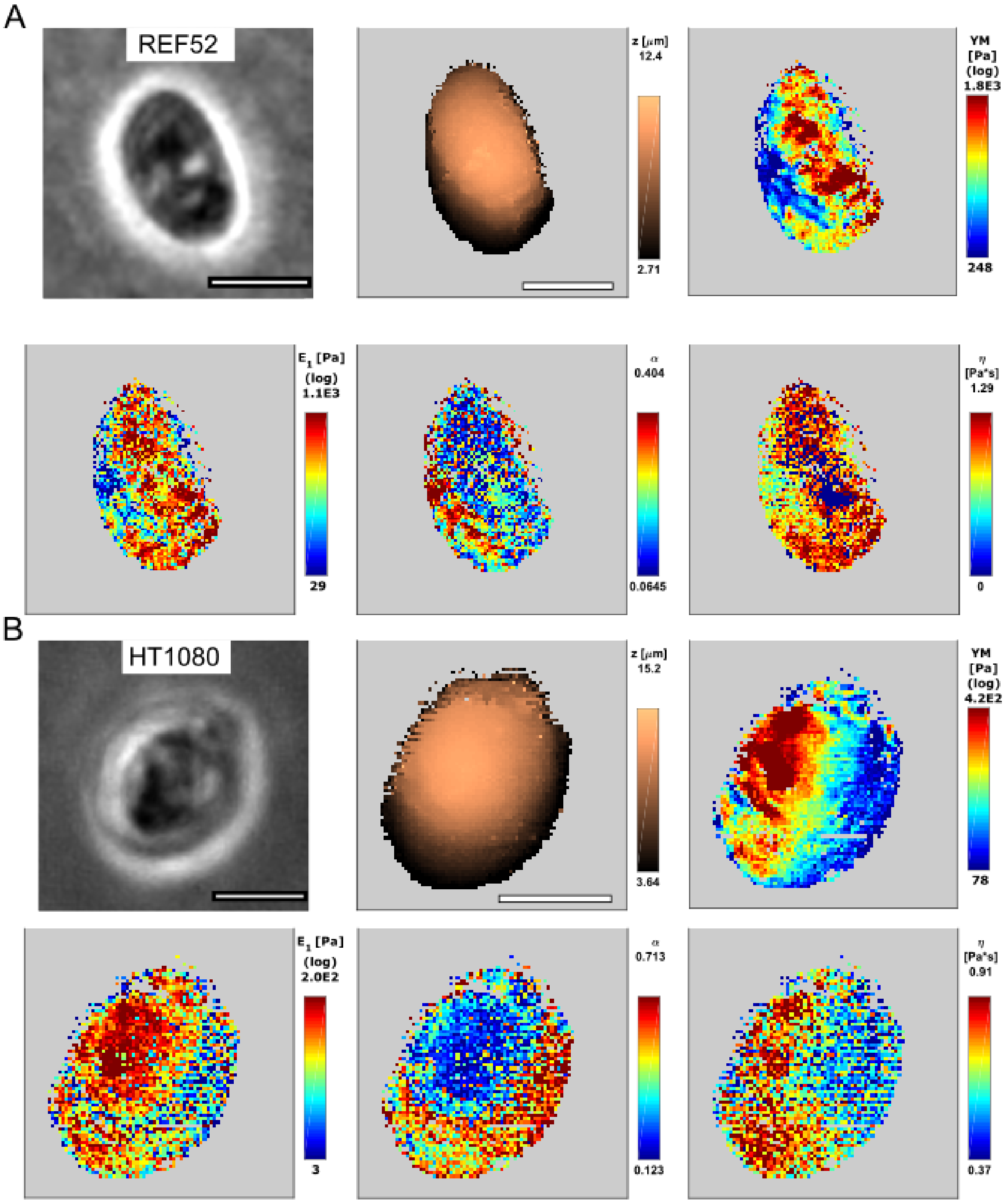

Figure S1. Examples of nanomechanical maps acquired over REF52 (A) and HT1080 (B) nucleoplasts: the topography (z), apparent YM, and viscoelastic parameters (E_1, α, η). Scale bars are 10 μm.
